# Cardiovascular magnetic resonance characterization of rheumatic mitral stenosis: findings from three worldwide endemic zones

**DOI:** 10.1186/s12968-022-00853-5

**Published:** 2022-04-07

**Authors:** Mahesh K. Vidula, Ziqian Xu, Yuanwei Xu, Abdullah Alturki, Bhavana N. Reddy, Prayaag Kini, Angel L. Alberto-Delgado, Ron Jacob, Tiffany Chen, Victor A. Ferrari, Lilia M. Sierra-Galan, Yucheng Chen, Sanjaya Viswamitra, Yuchi Han

**Affiliations:** 1 Division of Cardiovascular Medicine, Department of Medicine, Hospital of the University of Pennsylvania, Philadelphia, PA USA; 2 Department of Radiology, West China Hospital, Sichuan University, Chengdu, Sichuan China; 3 Division of Cardiology, Department of Medicine, West China Hospital, Sichuan University, Chengdu, Sichuan China; 4 Division of Cardiothoracic Imaging, Department of Radiology, Hospital of the University of Pennsylvania, Philadelphia, PA USA; 5 Department of Radiology, Sri Sathya Sai Institute of Higher Medical Sciences, Bangalore, Karnataka India; 6 Department of Cardiology, Sri Sathya Sai Institute of Higher Medical Sciences, Bangalore, Karnataka India; 7 Central Army Hospital of the National Secretary of Defense, Mexico City, Mexico; 8 Division of Cardiovascular Medicine, Lancaster General Hospital, Lancaster, PA USA; 9 Division of Cardiology, American British Cowdray Medical Center, Mexico City, Mexico; 10 Division of Cardiology, Biomedical Research Tower, The Ohio State University, Room 216, 460 W. 12th Avenue, Columbus, OH 43210 USA

**Keywords:** Rheumatic mitral stenosis, Cardiovascular magnetic resonance, Cardiac remodeling

## Abstract

**Background:**

Cardiac remodeling in rheumatic mitral stenosis (MS) is complex and incompletely understood. The objective of this study was to evaluate cardiac structural and functional changes in a cohort of patients with rheumatic MS using cardiovascular magnetic resonance (CMR).

**Methods:**

This retrospective study included 40 patients with rheumatic MS, consisting of 19 patients from India, 15 patients from China, and 6 patients from Mexico (median (interquartile range (IQR)) age: 45 years (34–55); 75% women). Twenty patients were included in the control group. CMR variables pertaining to morphology and function were collected. Late gadolinium enhancement (LGE) sequences were acquired for tissue characterization. Statistical analyses were performed using the Kruskal–Wallis test and the chi-square test.

**Results:**

Compared to the control group, patients with MS had lower left ventricular (LV) ejection fraction (51% (42%–55%) vs 60% (57%–65%), *p* < 0.001), lower right ventricular (RV) ejection fraction (44% (40%–52%) vs 64% (59%–67%), *p* < 0.001), higher RV end-diastolic volume (72 (58–87) mL/m^2^ vs 59 (49–69) mL/m^2^, *p* = 0.003), larger left atrial volume (87 (67–108) mL/m^2^ vs 29 (22–34) mL/m^2^, *p* < 0.001), and right atrial areas (20 (16–23) cm^2^ vs 13 (12–16) cm^2^, *p* < 0.001). LGE was prevalent in patients with rheumatic MS (82%), and was commonly located at the RV insertion sites. Furthermore, the patient cohorts from India, China, and Mexico were heterogeneous in terms of baseline characteristics and cardiac remodeling.

**Conclusion:**

Our findings demonstrated that biventricular dysfunction, right and left atrial remodeling, and LGE at the RV insertion sites are underappreciated in contemporary rheumatic MS. Further studies are needed to elucidate the prognostic implications of these findings.

**Supplementary Information:**

The online version contains supplementary material available at 10.1186/s12968-022-00853-5.

## Background

Rheumatic heart disease is the most common cause of mitral stenosis (MS), and remains a major cause of valve disease globally, occurring predominantly in developing countries [1–4]. Progressive MS leads to atrial arrhythmias, pulmonary hypertension, and heart failure, ultimately requiring percutaneous or surgical valve intervention [1, 5]. Studies utilizing echocardiography and cardiac catheterization have suggested that chronic MS results in adverse remodeling, leading to left atrial (LA) enlargement and dysfunction, pulmonary artery hypertension, and eventually right ventricular (RV) dilation and dysfunction [6]. While the left ventricle (LV) is typically thought to be spared from the adverse hemodynamic consequences of MS, LV dysfunction has been noted in patients with MS and is not well understood [7, 8]. Accurate and precise evaluation of cardiac morphology and function may ultimately help guide decisions regarding follow-up screening, further evaluation, and timing of intervention.

While echocardiography is typically the noninvasive modality of choice in the evaluation of patients with rheumatic MS due to its availability and low cost, cardiovascular magnetic resonance (CMR) imaging is a promising tool to provide more detailed characterization of morphologic and functional changes that occur in response to MS. CMR has the unique ability to precisely quantify chamber size and function due to its high spatial resolution, and can also provide tissue characterization through late gadolinium enhancement (LGE) imaging and parametric mapping. Prior studies have shown that CMR can improve the evaluation and prognostication of patients with other valve diseases, such as aortic stenosis, mitral regurgitation, and tricuspid regurgitation, through more accurate assessment of cardiac remodeling and tissue characterization [9–11]. However, due to the limited availability of CMR in countries with a high prevalence of rheumatic MS, very few CMR studies have been performed in patients with MS. While CMR has primarily been utilized in small cohorts to assess mitral valve area and transmitral gradients, only one small study examined LV remodeling after balloon valvuloplasty and no studies to date have performed tissue mapping sequences in this population [12–17]. Thus, the current understanding of cardiac remodeling and tissue characteristics in patients with rheumatic MS remains incomplete.

In this study, we assembled a cohort of patients with rheumatic MS who underwent CMR at one of three endemic countries on two continents, and characterized the cardiac structural and functional changes in this population.

## Methods

### Study population

We studied consecutive patients with symptomatic rheumatic MS referred for CMR in China between 2013 and 2019 (West China Hospital, Sichuan University, Chengdu, Sichuan Province, China), India between 2018 and 2020 (Sri Sathya Sai Institute of Higher Medical Sciences, Bangalore, Karnataka, India), and Mexico between 2012 and 2020 (American British Cowdray Medical Center, Mexico City, Mexico) (n = 76). Patients in India underwent CMR as part of a part of a study protocol, patients in Mexico underwent CMR as part of either a study protocol or preoperative evaluation, and patients in China underwent CMR as part of a preoperative evaluation. We excluded patients with prior balloon valvuloplasty, prior cardiovascular procedures, prior myocardial infarction, or greater than moderate concomitant valvular disease as assessed by echocardiography. The control group was derived from a cohort in India that underwent CMR for the evaluation of coronary artery disease (CAD). These patients did not have any abnormalities on rest imaging or stress perfusion imaging, did not have any known cardiovascular conditions, and did not have a history of prior percutaneous coronary intervention or coronary artery bypass graft surgery. This study received approval from each hospital’s respective Institutional Review Board, and patients gave written informed consent for the study.

### Cardiovascular magnetic resonance protocol and analysis

The following CMR scanners were utilized in this study: (1) India: 1.5 T (Aera, Siemens Healthineers, Erlangen, Germany), (2) China: 3 T (Trio or Skyra, Siemens Healthineers), and (3) Mexico: 1.5 T (Achieva, Philips Heathcare, Best, the Netherlands). At all three sites, after acquiring localizers, electrocardiogram gated standard balanced steady-state free precession cine images with 8 to 14 short-axis and 3 long-axis planes (2-, 3-, and 4-chamber views) were obtained. The parameters were as follows: (1) India: Repetition time (TR)/Echo time (TE) 4.4–5.4/1.1–1.2 ms; slice thickness: 8 mm; flip angle: 66–67 degrees; in-plane spatial resolution: 1.4–1.7 × 1.7–2.3 mm; (2) China: TR/TE, 2.8 /1.2 ms; slice thickness: 8 mm; flip angle: 38 degrees; in-plane spatial resolution: 1.6 × 2.0 mm; (3) Mexico: TR/TE 3.7–3.9/1.8–1.9 ms; slice thickness: 8 mm; flip angle: 60 degrees; in-plane spatial resolution: 1.6–1.7 × 1.6–1.7 mm. Ten to 20 minutes after the administration of a gadolinium contrast agent, segmented two-dimensional LGE images were acquired using routine inversion recovery sequences with the following parameters: (1) India: TE 1.1 ms; slice thickness: 8 mm; flip angle: 40 degrees; field of view: 274 × 319 mm; in-plane spatial resolution: 1.2 × 2.1 mm; (2) China: TE 1.2 ms; slice thickness: 8 mm; flip angle: 40 degrees; field of view: 225 × 300 mm; in-plane spatial resolution: 2.1 × 1.6 mm; (3) Mexico: TE 3.0 ms; slice thickness: 10 mm; flip angle: 25 degrees; field of view: 360–460 × 360–460 mm; in-plane spatial resolution: 1.3–1.6 × 1.6–2.1 mm. All studies included in the analysis were judged to be of good quality. CMR studies from India and Mexico were analyzed at the coordinating center (University of Pennsylvania, Philadelphia, Pennsylvania, USA) by two investigators. CMR studies from China were analyzed locally due to export restrictions, but LGE images were virtually reviewed together with the coordinating center. Measurements of biventricular volumes and function, LA volume (using biplane area-length method) and emptying fraction, right atrial (RA) maximal area, and mitral valve area (MVA) by planimetry were performed, and inter-observer and intra-observer variability analyses were conducted. Papillary muscles were included in the LV cavity, not in the LV mass. Native T1 measurements and quantification of extracellular volume fraction (ECV) were performed on a portion of CMR studies from India (n = 18) and China (n = 9). Wall motion abnormalities were assessed by reviewing cine images, and LGE images were analyzed for the presence and location of LGE. CMR studies from India and Mexico were analyzed using suiteHEART (version 5.0.1, NeoSoft, Milwuakee, Wisconsin, USA). CMR studies from China were analyzed on Qmass (version 8.1, Medis, Leiden, the Netherlands.

### Baseline characteristics

Baseline clinical characteristics (demographics, comorbidities, vital signs, body surface area, and prior cardiac procedures, including prior valvuloplasty) at the time of the CMR were obtained from the medical record. N-terminal pro brain natriuretic (NTpro-BNP) levels were available for the cohort from India. MVA, as determined by pressure half-time (PHT), tricuspid regurgitation peak velocity for determination of pulmonary artery systolic pressure and other concomitant valve diseases were extracted from the transthoracic echocardiogram report from the study performed closest to the time of the CMR. Severe MS was defined as MVA ≤ 1.5 cm^2^.

### Statistical analysis

Non-normally distributed continuous variables are shown as median (interquartile range) and analyzed using the Kruskal–Wallis test. Categorical variables are shown as total counts with percentages and analyzed using the chi-square test. All statistical analyses were performed with JMP Pro (version 15, SAS Institute, Cary, North Carolina, USA).

## Results

### Baseline characteristics

**Fig. 1:**
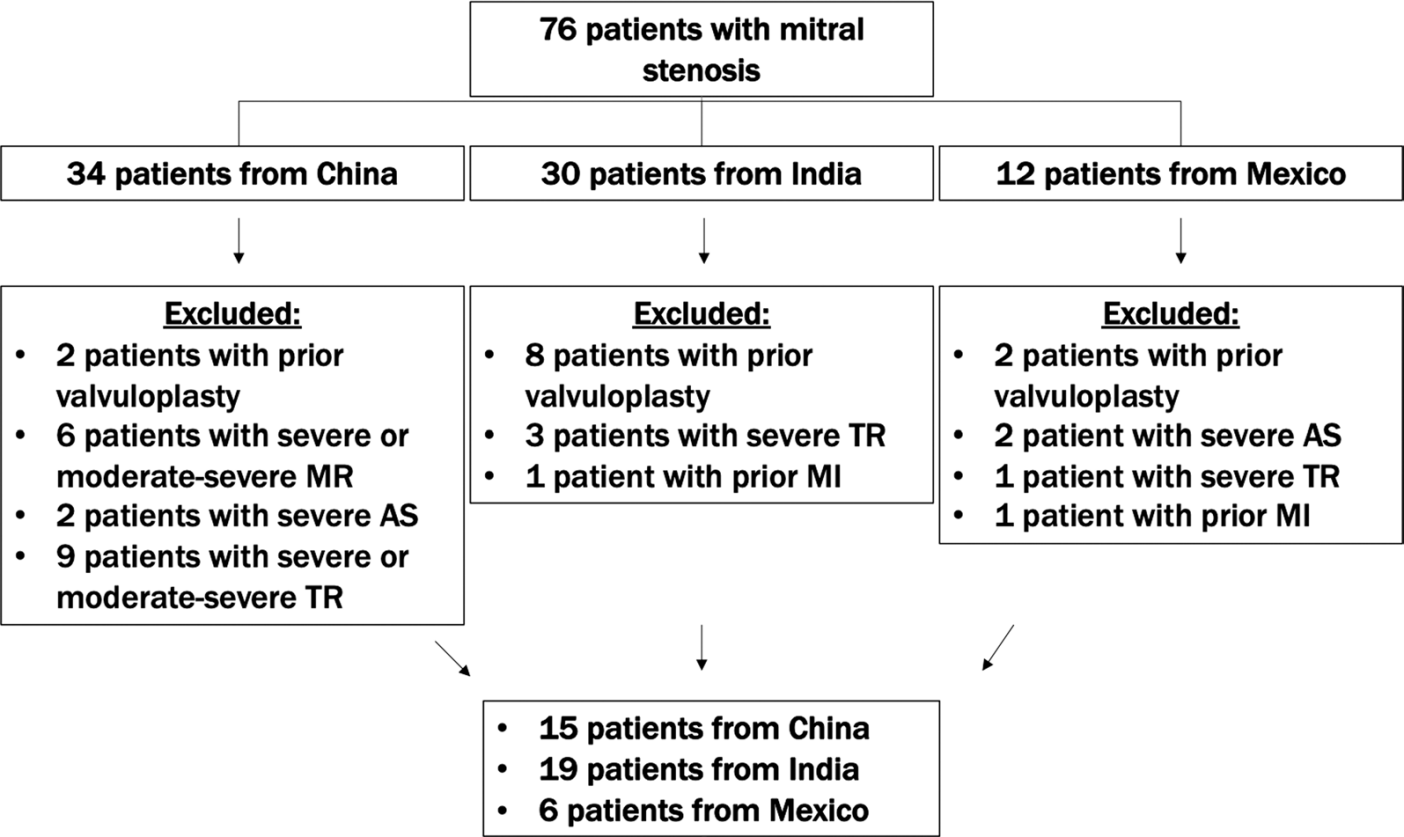
Flow diagram of patients included in the analysis. AS, aortic stenosis; MI, myocardial infarction; MR, mitral regurgitation; TR, tricuspid regurgitation

The final study cohort consisted of 40 patients, including 19 patients from India, 15 patients from China, and 6 patients from Mexico (Fig. 1). Characteristics of the overall MS cohort and the control group are shown in Table 1. Patients with MS were younger (median 45 years, interquartile range (IQR): 34 to 55 years) compared to the control group (median: 54 years, IQR: 45 to 62 years, *p* = 0.03). The control group was derived from a cohort in India that underwent CMR for the evaluation of CAD, and patients in the control group had higher rates of CAD, diabetes mellitus, and hypertension. The MS cohort reported a higher burden of symptoms, including dyspnea on exertion, palpitations, and angina. Echocardiography data for the calculation of MVA by PHT was available for the majority of the overall cohort (4/15 patients in the China cohort, 18/19 patients in the India cohort, and 6/6 patients in the Mexico cohort). Within the context of this limitation, most patients in the MS cohort were classified as severe MS, as indicated by the median MVA by PHT of 0.85 cm^2^ (IQR: 0.73 to 1.10 cm^2^) and median MVA by CMR planimetry of 1.17 cm^2^ (IQR: 0.89 to 1.56 cm^2^). The patients in the MS cohort had the following breakdown of coexistent mitral regurgitation: none (12/40 [30%]), trace (7/40 [17.5%]), mild (11/40 [27.5%]), mild-moderate (1/40 [2.5%]), and moderate (9/40 [22.5%]).

**Table 1:** Clinical characteristics and CMR findings of the mitral stenosis cohort and controls

Characteristics	Mitral stenosis (n = 40)	Controls (n = 20)	*P* value
*Demographics*			
Age	45 (34, 55)	54 (45, 62)	0.03
Female	30 (75%)	12 (60%)	0.23
*Comorbidities*			
Coronary artery disease	0 (0%)	6 (30%)	< 0.001
Atrial fibrillation	17 (43%)	0 (0%)	< 0.001
Diabetes mellitus	3 (8%)	6 (30%)	0.02
Hypertension	5 (13%)	10 (50%)	0.002
Mitral valve area by PHT on echocardiography (cm^2^)	(n = 28) 0.85 (0.73, 1.10)	Not applicable	Not applicable
*Symptoms prior to CMR*			
Dyspnea on exertion	24 (60%)	2 (10%)	< 0.001
Palpitations	17 (43%)	0 (0%)	< 0.001
Syncope	3 (8%)	1 (5%)	0.71
Angina	18 (46%)	2 (10%)	0.006
*Vital signs prior to CMR*			
Systolic blood pressure (mmHg)	115 (108, 127)	130 (128, 150)	< 0.001
Diastolic blood pressure (mmHg)	72 (67, 79)	80 (70, 90)	0.02
Heart rate (beats per minute)	80 (67, 99)	76 (56, 81)	0.03
Body surface area (m^2^)	1.60 (1.49, 1.70)	1.70 (1.58, 1.79)	0.01
*CMR findings*			
Mitral valve area by planimetry (cm^2^)	1.17 (0.89, 1.56)	4.05 (3.73, 4.5)	< 0.001
LA maximal volume (mL/m^2^)	87 (67, 108)	29 (22, 34)	< 0.001
LA minimal volume (mL/m^2^)	68 (52, 88)	11 (8, 14)	< 0.001
LA emptying fraction (%)	20 (13, 30)	61 (51, 65)	< 0.001
RA maximal area (cm^2^)	20 (16, 23)	13 (12, 16)	< 0.001
LVEF (%)	51 (42, 55)	60 (57, 65)	< 0.001
LVEDV (mL)	114 (97, 151)	113 (94, 132)	0.48
LVEDVI (mL/m^2^)	72 (61, 93)	68 (57, 80)	0.17
LVESV (mL)	57 (43, 79)	40 (35, 59)	0.02
LVSVI (mL/m^2^)	35 (27, 49)	26 (20, 34)	0.004
LV stroke volume index (mL/m^2^)	36 (28, 44)	40 (36, 47)	0.16
Cardiac index (L/min/m^2^)	2.8 (2.4, 3.4)	2.8 (2.1, 3.5)	0.89
LV mass index (g/m^2^)	43 (34, 52)	33 (29, 44)	0.02
LV global longitudinal strain (%)	(n = 20) − 16 (− 18, − 14)	(n = 20) − 20 (− 22, − 18)	< 0.001
RVEF (%)	44 (40, 52)	64 (59, 67)	< 0.001
RVEDV (mL)	113 (98, 142)	93 (86, 116)	0.02
RVEDVI	72 (58, 87)	59 (49, 69)	0.003
RVESV (mL)	66 (46, 80)	33 (30, 39)	< 0.001
RVESVI	41 (29, 48)	19 (18, 23)	< 0.001
Wall motion abnormalities	0 (0%)	0 (0%)	NS
LGE presence	(n = 39) 32 (82%)	(n = 20) 1 (5%)	< 0.001
Epicardial	0%	0%	NS
Midmyocardial	32 (82%)	1 (5%)	< 0.001
Subendocardial	0 (0%)	0 (0%)	NS
LGE – inferior RV insertion	31 (79%)	1 (5%)	< 0.001
LGE – superior RV insertion	5 (13%)	0 (0%)	0.006

Data are shown as median (interquartile range) or n (%). CMR, cardiovascular magnetic resonance; LA, left atrium; LGE, late gadolinium enhancement; LVEDV, left ventricular end-diastolic volume; LVEDVI left ventricular end-diastolic volume index; LVEF, left ventricular ejection fraction; LVESV, left ventricular end-systolic volume; LVESVI, left ventricular end-systolic volume index; LV, left ventricle; MS, mitral stenosis; MVA, mitral valve area; NS, not significant; PHT, pressure half-time; RA, right atrium; RV, right ventricle; RVEDV, right ventricular end-diastolic volume; RVEDVI, right ventricular end-diastolic volume index; RVEF, right ventricular ejection fraction; RVESV, right ventricular end-systolic volume; RVESVI, right ventricular end-systolic volume index

Characteristics of the MS cohort, stratified by country, are shown in Table 2. The India cohort was younger (median 34 years, IQR: 24 to 41 years), compared to the China cohort (median 55 years, IQR: 52 to 64 years) and Mexico cohort (median 53, IQR: 48 to 65 years, *p* < 0.001). Patients in China had higher rates of atrial fibrillation, and patients in Mexico had higher rates of hypertension. Furthermore, there was a higher burden of dyspnea on exertion in the India and Mexico groups, and a higher burden of angina in the China and Mexico groups. There were no significant differences in MVA by PHT or CMR planimetry between the three groups.

**Table 2:** Clinical characteristics and CMR findings of the mitral stenosis cohort, stratified by country

Characteristics	China (n = 15)	India (n = 19)	Mexico (n = 6)	*P* value
*Demographics*				
Age (years)	55 (52, 64)	34 (24, 41)	53 (48, 65)	< 0.001
Female	12 (80%)	12 (63%)	6 (100%)	0.16
*Comorbidities*				
Coronary artery disease	0 (0%)	0 (0%)	0 (0%)	NS
Atrial fibrillation	11 (73%)	4 (21%)	2 (33%)	0.008
Diabetes	2 (13%)	0 (0%)	1 (17%)	0.22
Hypertension	2 (13%)	0 (0%)	3 (50%)	0.005
Mitral valve area by PHT on echocardiogram (cm^2^)	(n = 4) 1.3 (0.7, 1.9)	(n = 18) 0.8 (0.7, 1)	(n = 6) 1.2 (0.9, 1.4)	0.16
PASP on echocardiogram (mmHg)	–	43 (30, 53)	38 (32, 42)	0.33
*Symptoms prior to CMR*				
Dyspnea on exertion	3 (20%)	15 (79%)	6 (100%)	< 0.001
Palpitations	4 (27%)	9 (47%)	4 (67%)	0.21
Syncope	2 (13%)	0 (0%)	1 (17%)	0.22
Angina	14 (100%)	0 (0%)	4 (67%)	< 0.001
*Vital signs prior to CMR*				
Systolic blood pressure (mmHg)	110 (107, 118)	116 (112, 128)	121 (106, 135)	0.52
Diastolic blood pressure (mmHg)	72 (69, 79)	72 (62, 80)	74 (51, 84)	0.79
Heart rate (beats per minute)	84 (74, 101)	80 (60, 103)	75 (66, 89)	0.64
Body surface area (m^2^)	1.63 (1.53, 1.72)	1.56 (1.47, 1.66)	1.72 (1.51, 1.80)	0.09
*CMR findings*				
Mitral valve area by planimetry (cm^2^)	(n = 12) 1.9 (1.4, 2.2)	(n = 19) 1 (0.7, 1.2)	(n = 6) 1.3 (0.9, 1.6)	< 0.004
LA maximal volume (mL/m^2^)	109 (90, 153)	80 (59, 98)	80 (68, 98)	0.008
LA minimal volume (mL/m^2^)	95 (63, 139)	59 (37, 83)	57 (52, 81)	0.03
LA emptying fraction (%)	17 (11, 30)	20 (13, 33)	24 (18, 27)	0.57
RA maximal area (cm^2^)	22 (16, 25)	17 (15, 22)	21 (19, 23)	0.12
LVEF (%)	48 (40, 56)	48 (41, 52)	63 (54, 67)	0.007
LVEDV (mL)	133 (103, 167)	105 (88, 136)	123 (106, 144)	0.24
LVEDVI (mL/m^2^)	74 (66, 94)	64 (58, 93)	72 (64, 92)	0.46
LVESV (mL)	76 (46, 93)	57 (45, 61)	42 (38, 64)	0.18
LVESVI (mL/m^2^)	43 (27, 51)	35 (31, 42)	27 (23, 36)	0.20
LV stroke volume index (mL/m^2^)	35 (27, 44)	33 (27, 42)	46 (38, 53)	0.08
Cardiac index (L/min/m^2^)	3.2 (2.6, 3.4)	2.5 (2.2, 3.5)	3.1 (2.6, 4.0)	0.26
LV mass index (g/m^2^)	50 (42, 63)	35 (30, 46)	44 (35, 54)	0.003
LV global longitudinal strain (%)	Not performed	(n = 14) − 16 (− 17, − 14)	(n = 6) − 16 (− 18, − 14)	0.62
RVEF (%)	43 (40, 54)	43 (36, 49)	49 (44, 59)	0.15
RVEDV (mL)	110 (86, 128)	124 (104, 147)	111 (96, 153)	0.32
RVEDVI (mL/m^2^)	65 (53, 77)	76 (68, 89)	72 (55, 89)	0.09
RVESV (mL)	61 (45, 73)	68 (59, 88)	57 (40, 89)	0.35
RVESVI (mL/m^2^)	35 (28, 46)	42 (40, 55)	36 (23, 51)	0.11
Wall motion abnormalities	0 (0%)	0 (0%)	0 (0%)	NS
LGE presence	(n = 14) 11 (79%)	(n = 19) 15 (79%)	(n = 6) 6 (100%)	0.46
Epicardial	0%	0%	0%	NS
Midmyocardial	11 (79%)	15 (79%)	6 (100%)	0.46
Subendocardial	0 (0%)	0 (0%)	0 (0%)	NS
LGE – inferior RV insertion	11 (79%)	15 (79%)	5 (83%)	0.97
LGE – superior RV insertion	4 (29%)	0 (0%)	1 (17%)	< 0.001
Native T1 (ms)	(n = 9) 3 T scanner: 1275 (1236, 1340)	(n = 18) 1.5 T scanner: 990 (964, 1029)	–	Not applicable*
ECV (%)	(n = 8) 28.6 (28.1, 29.4)	(n = 17) 27.3 (23.3, 30.4)	–	0.43

Data are shown as median (interquartile range) or n (%). CMR, cardiovascular magnetic resonance; LA, left atrium; LGE, late gadolinium enhancement; LVEDV, left ventricular end-diastolic volume; LVEDVI left ventricular end-diastolic volume index; LVEF, left ventricular ejection fraction; LVESV, left ventricular end-systolic volume; LVESVI, left ventricular end-systolic volume index; LV, left ventricle; MS, mitral stenosis; MVA, mitral valve area; NS, not significant; PHT, pressure half-time; RA, right atrium; RV, right ventricle; RVEDV, right ventricular end-diastolic volume; RVEDVI, right ventricular end-diastolic volume index; RVEF, right ventricular ejection fraction; RVESV, right ventricular end-systolic volume; RVESVI, right ventricular end-systolic volume index

^*^Native T1 values performed on 3 T and 1.5 T scanners cannot be directly compared

### CMR findings in the overall mitral stenosis cohort and control groups

**Fig. 2:**
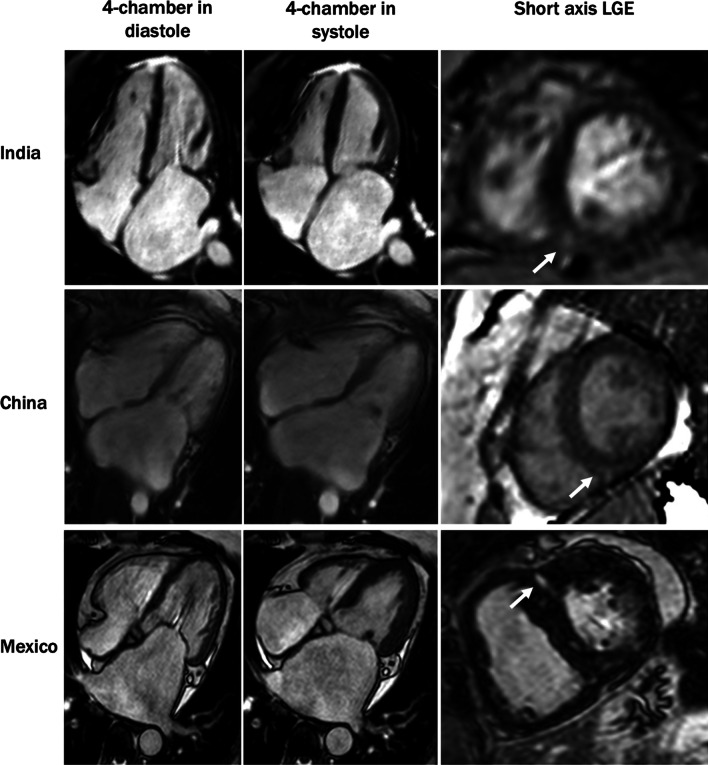
Representative examples of cardiovascular magnetic resonance images of patients with rheumatic mitral stenosis from India, China, and Mexico. White arrows point to regions of late gadolinium enhancement (LGE) at the right ventricular (RV) insertion sites

The CMR findings in the MS cohort and the control group are shown in Table 1. Inter-observer and intra-observer variability of these measurements was low (Additional file 1: Table S1). Representative images of CMR findings from each country are shown in Fig. 2.

Compared to the control group, the MS patient cohort had significantly lower LV ejection fraction (LVEF) (51% (IQR: 42% to 55%) vs 60% (IQR: 57% to 65%), *p* = 0.001), and RV ejection fraction (RVEF) (44% (IQR: 40% to 52%) vs 64% (IQR: 59% to 67%), *p* < 0.001). RV volumes were greater in the MS cohort (end-diastolic volume index (RVEDVI) of 72 (IQR: 58 to 87) mL/m^2^ vs 59 (IQR: 49 to 69) mL/m^2^, *p* = 0.003), and there was no difference in LV end-diastolic index (LVEDVI) between the two groups. LV global longitudinal strain (GLS) was worse (less negative) in the MS cohort (− 16% (IQR: − 18% to − 14%) vs − 20% (IQR: − 22% to − 18%), *p* < 0.001). Pulmonary artery systolic pressures (PASP) by transthoracic echocardiography were mildly to moderately elevated in the MS cohort. In the MS cohort, there was a weak negative correlation between PASP and RVEF (r = − 0.43, *p* = 0.03) and PASP and RV stroke volume (SV)/end systolic volume (RVESV) (r = − 0.49, *p* = 0.02), but no correlation was observed between PASP and RV diastolic or systolic volumes.

Indexed LA volumes were three times greater in the MS cohort than in the control group (median LA maximal volume of 87 (IQR: 67 to 108) mL/m^2^ vs 29 (IQR: 22 to 34) mL/m^2^, *p* < 0.001), and LA emptying fraction was significantly lower in the MS group (20% (IQR: 13% to 30%) vs 61% (IQR: 51% to 65%), *p* < 0.001). RA size was larger in the MS group (20 (IQR: 16 to 23) cm^2^ vs 13 (IQR: 12 to 16) cm^2^, p < 0.001). There were no significant correlations between MVA by echocardiography, biatrial or biventricular size/function, or PASP (Additional file 1: Table S2).

**Fig. 3:**
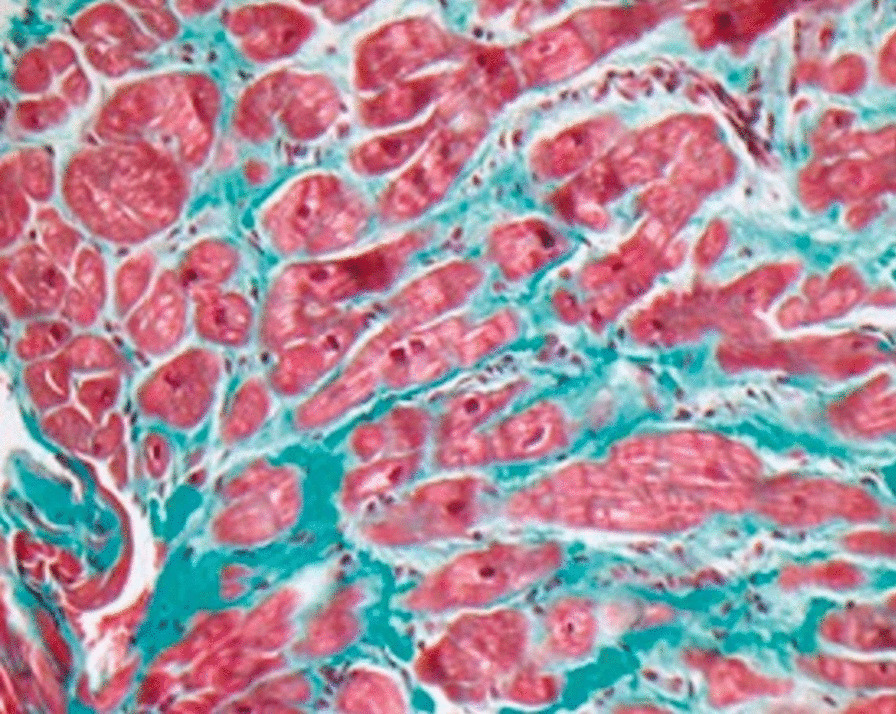
Endomyocardial biopsy from a mitral stenosis patient in Mexico. Biopsy was performed at the area of LGE involving the RV insertion site and revealed myocardial fibrosis, demonstrating correlation between LGE and histology

LGE sequences were acquired in 39/40 of the patients in the MS cohort, and in all patients in the control group. The presence of LGE was more common in the MS cohort (82% vs 5%, *p* < 0.001). In the MS group, the two patterns of LGE observed were 1) enhancement at the inferior RV insertion site (79%) and 2) enhancement at the superior RV insertion site (13%). There were no patients with subepicardial or subendocardial LGE. One patient in the control group exhibited midmyocardial enhancement at the inferior RV insertion site. There were no patients with wall motion abnormalities in either group. One patient in Mexico underwent endomyocardial biopsy at the RV insertion site that revealed myocardial fibrosis (Fig. 3).

There were no significant differences in CMR characteristics between patients in the MS cohort, when stratified by the presence of dyspnea on exertion or palpitations. However, maximal LA volumes and maximal RA areas were greater in MS patients with angina than in those without angina (Additional file 1: Table S3). In addition, there were no significant differences in CMR characteristics when patients in the MS cohort were stratified by presence of atrial fibrillation (Table 3) or LGE (Additional file 1: Table S4).

**Table 3:** CMR findings of the mitral stenosis (MS) cohort, stratified by atrial fibrillation

	MS patients with atrial fibrillation (n = 17)	MS patients without atrial fibrillation (n = 23)	*P* value
LA maximal volume (mL/m^2^)	98 (83, 136)	80 (64, 103)	0.09
LA minimal volume (mL/m^2^)	83 (58, 107)	63 (45, 85)	0.12
LA emptying fraction (%)	19 (11, 28)	20 (14, 33)	0.47
RA maximal area (cm^2^)	22 (17, 24)	20 (15, 22)	0.33
LVEF (%)	48 (42, 63)	51 (41, 54)	0.49
LVEDV (mL/m^2^)	73 (58, 90)	68 (61, 93)	0.68
RVEF (%)	43 (39, 54)	45 (40, 50)	0.89
RVEDVI (mL/m^2^)	69 (58, 78)	76 (61, 89)	0.26
LGE presence	(n = 16) 13 (81%)	(n = 23) 19 (83%)	0.91
MVA by pressure half-time on echocardiography (cm^2^)	(n = 10) 0.9 (0.7, 1.1)	(n = 18) 0.8 (0.75, 1.3)	0.98

Data are shown as median (interquartile range) or n (%)

Data are shown as median (interquartile range) or n (%). CMR, cardiovascular magnetic resonance; LA, left atrium; LGE, late gadolinium enhancement; LVEDV, left ventricular end-diastolic volume; LVEF, left ventricular ejection fraction; MS, mitral stenosis; MVA, mitral valve area; RA, right atrium; RV, right ventricle; RVEDVI, right ventricular end-diastolic volume index; RVEF, right ventricular ejection fraction

### Differences in CMR findings between the MS cohorts from China, India, and Mexico

The CMR findings in the MS cohort, stratified by country, are shown in Table 2. Notably, patients from China and India had significantly lower LV function than patients from Mexico (*p* = 0.007). The China and India cohorts had a mildly reduced median LVEF of 48%, while the Mexico group had a normal median LVEF of 63%. RV function was reduced across all three groups, and biventricular volumes were similar in the three cohorts. There was also no difference in LV GLS between the India and Mexico cohorts.

Indexed LA volumes were significantly greater in the China cohort (median: 109 (IQR: 90 to 153) mL/m^2^) compared to the India (80 (IQR: 59 to 98) mL/m^2^) and Mexico (80 (IQR: 68 to 98) mL/m^2^) cohorts, but LA emptying fraction was similar in all groups. RA maximal areas were similar across the three cohorts.

The prevalence of LGE was similar across all three MS cohorts. There was no difference in the frequency of midmyocardial enhancement at the inferior RV insertion point, but midmyocardial LGE at the superior RV insertion point was more commonly observed in the China group. In the India cohort, the median native T1 time (at 1.5 T) (n = 18) was 990 ms (IQR: 964 to 1029 ms). In the China cohort, the median native T1 time (at 3 T) (n = 9) was 1275 ms (IQR: 1236 to 1340 ms). The median ECV in both cohorts was 28.3% (IQR: 24.5% to 29.9%), and there was no difference in ECV between the India and China groups (27.3% (IQR: 23.3% to 30.4%) vs 28.9% (IQR: 28.1% to 29.4%), *p* = 0.43). Patients with LVEF ≤ 50% had similar ECV when compared to patients with LVEF ≥ 50% (ECV: 28.5% (IQR: 25.6% to 30.2%) vs 28.0% (IQR: 23.5% to 29.7%), *p* = 0.41).

NT-proBNP levels were available for the India cohort. In this group, NT-proBNP had significant positive correlations with RV ESVI (r = 0.81, *p* < 0.001) and RVEDVI (r = 0.55, *p* = 0.02), and significant negative correlations with RVEF (r = − 0.81, *p* < 0.001), RV SV/ESV (r = − 0.69, *p* = 0.002), LVEF (r = − 0.60, *p* = 0.01), and LA emptying fraction (r = − 0.60, *p* = 0.01) (Table 4).

**Table 4:** Correlations between NT-proBNP and CMR parameters in the mitral stenosis cohort

	Correlation coefficient (r)	P value
LA emptying fraction	− 0.60	0.01
LA maximal volume (mL/m^2^)	0.08	0.77
LVEF (%)	− 0.60	0.01
LVEDV (mL)	− 0.35	0.17
LVEDVI (mL/m^2^)	− 0.32	0.20
LVESV (mL)	− 0.11	0.67
LVESVI (mL/m^2^)	− 0.06	0.82
LVSV (mL)	− 0.53	0.03
RVEF (%)	− 0.81	< 0.001
RVEDV (mL)	0.41	0.10
RVEDVI	0.55	0.02
RVESV (mL)	0.74	< 0.001
RVESVI	0.81	< 0.001
RV SV/ESV	− 0.69	0.002

Data are shown as median (interquartile range) or n (%). CMR, cardiovascular magnetic resonance; LA, left atrium; LVEDV, left ventricular end-diastolic volume; LVEDVI left ventricular end-diastolic volume index; LVEF, left ventricular ejection fraction; LVESV, left ventricular end-systolic volume; LVESVI, left ventricular end-systolic volume index; RVEDV, right ventricular end-diastolic volume; RVEDVI, right ventricular end-diastolic volume index; RVEF, right ventricular ejection fraction; RVESV, right ventricular end-systolic volume; RVESVI, right ventricular end-systolic volume index

Overall, 22/38 (58%) patients with MS ultimately underwent a mitral valvuloplasty or valve replacement (10/13 from China, 8/19 from India, and 4/6 from Mexico). The characteristics of patients, stratified by whether a mitral valve procedure was performed, are described in Additional file 1: Table S5. In general, patients who underwent a mitral valve intervention were older, had higher rates of angina, and had smaller RV volumes. Patients who did not undergo a valve intervention had worse (less negative) LV GLS. There were no significant differences in CMR characteristics between patients who underwent valve replacement or valvuloplasty (Additional file 1: Table S6).

## Discussion

In this study of a cohort of patients from China, India, and Mexico with rheumatic MS who underwent CMR imaging, we assessed detailed cardiac structural and functional changes that occurred in response to MS. We found that biventricular and biatrial remodeling was common in this population. We also identified a high prevalence of nonischemic fibrosis at the RV insertion points in the overall cohort, but no significant elevation in native T1 or ECV. In addition, we found notable differences in cardiac structure and function between patients from the three countries.

As MS progresses and becomes more severe, the resistance to blood flow through the narrowed mitral valve orifice results in LA pressure elevation leading to LA enlargement and functional decline, pulmonary hypertension, and ultimately RV dilation and dysfunction [18]. Accordingly, in this analysis, we found that patients with rheumatic MS exhibited enlarged LA volumes and poor LA function, likely in part due to increased LA pressures. Notably, LA volumes were severely enlarged, and three times greater in size, in patients with MS compared to controls. Similarly, we found that patients with MS had increased RV volumes and reduced RV function compared to controls, which was also likely associated with the increased RA sizes seen in the MS cohort. Furthermore, we found a weak but significant negative correlation between PASP and RV systolic function, suggesting that the increased PA pressures that result from MS may lead to reductions in RV function. Understanding how these cardiac chambers remodel after valve intervention, and whether pre-procedural or post-procedural remodeling is associated with outcomes, should be the target of future studies. Furthermore, we found that NT-proBNP levels strongly correlated with RV volumes and markers of RV function. Since elevated NT-proBNP levels may indicate late stage and more severe RV disease, and RV evaluation by echocardiography can be challenging, these findings suggest that CMR may be able to provide a more complete evaluation of RV performance in patients with MS.

Interestingly, in the overall cohort, LV function was more reduced among MS patients, as assessed by LVEF and GLS. Although the LV was traditionally thought to be spared from adverse hemodynamic consequences due to MS, LV dysfunction in patients with MS has been recognized [7, 17].The mechanism of LV dysfunction in patients with rheumatic MS is uncertain. Prior studies have postulated a variety of mechanisms of LV dysfunction, including reduced preload [19], reduced LV filling during diastole [7], and increased afterload without compensatory increase in preload due to the MS [22]. Reduced LV filling is controversial since studies have shown conflicting results regarding whether there is a significant increase in end-diastolic volumes following valvuloplasty [20, 21]. Other mechanisms that have been proposed include chronic inflammation resulting in myocardial fibrosis [7], thickening and scarring of the subvalvular apparatus and subsequent wall motion abnormalities [7], and abnormal septal motion secondary to RV dysfunction. [23].

Our CMR study provides the opportunity to better understand the mechanisms related to LV dysfunction in MS. We found LVEDVs were similar in the MS and control groups, and we did not identify segmental wall motion abnormalities in the MS cohort. LGE imaging showed evidence of inferior and superior RV insertion site fibrosis, which has not been shown previously, and was likely due to RV remodeling in the setting of pulmonary artery hypertension [24–26]. Importantly, LGE imaging revealed no other nonischemic or ischemic etiologies of myocardial injury, such as myocarditis or prior infarction. Parametric mapping in the India and China cohorts demonstrated no increase in native T1 or ECV, even after stratifying by LVEF, suggesting lack of significant diffuse fibrosis to explain the reduced LV function observed in MS patients. These findings suggest that rheumatic inflammation may *not* be a significant cause of subsequent myocardial dysfunction in MS patients. Given the burden of RV dilation and dysfunction in this cohort, it is possible that LV dysfunction is a result of abnormal septal motion and altered RV-LV interactions. In addition, biventricular dysfunction could be a consequence of a tachycardia-mediated cardiomyopathy in the setting of atrial fibrillation. Additional work is required to understand the mechanisms and prognostic implications of LV dysfunction and LGE in this cohort. The indexed LV volumes were at the lower limits of normal, possibly due to reduced preload or reduced filling, but this requires additional study [27].

There were several key differences between the China, India, and Mexico cohorts. The India cohort was younger than the China and Mexico cohorts, suggesting that advanced valvular disease and subsequent heart failure symptoms developed more rapidly in this cohort. Prior studies have noted that patients in endemic regions tend to present with more rapidly progressive valve disease, possibly due to recurrent bouts of carditis [5]. Furthermore, patients in India and China exhibited greater LV dysfunction than the patients in Mexico, who had normal LV function. While these differences may be attributable to heterogeneity in progression of rheumatic heart disease in the three countries, differences in access to health care may also explain the more advanced presentations seen in India and China.

This study should be interpreted in the context of its strengths and limitations. Strengths include the careful selection of patients with rheumatic MS without concomitant valve disease and cardiovascular conditions, who underwent CMR across three countries. We were able to identify a sizable cohort of patients meeting our stringent criteria, despite the limited access and availability of CMR in these countries. In addition, tissue characterization was performed in the majority of CMR studies.

## Limitations

Our study has a number of limitations. Only patients undergoing preoperative CMR were included which reflect a selection bias. Despite this limitation, these patients represent a cohort of patients with symptomatic rheumatic MS. Native T1 mapping was not available on all scans. T2 mapping was not performed, and acute inflammation and edema could not be assessed. However, in general, MS is a late finding in patients with rheumatic heart disease, and patients are typically decades past the acute inflammatory carditis/valvulitis stage. Finally, outcome data are not available and therefore associations of imaging parameters with clinical outcomes could not be performed.

## Conclusions

In this multicenter international study of patients with symptomatic rheumatic MS, we found that biventricular and biatrial remodeling, including reduced biventricular function and nonischemic fibrosis at the RV insertion sites. In addition, the patient cohorts from India, China, and Mexico were heterogeneous in terms of baseline characteristics and ventricular remodeling, possibly due to differences in disease progression and access to care. These findings further our understanding of the cardiac structural and functional changes in rheumatic MS, and demonstrate the value of CMR imaging in the evaluation of this population. Further studies are needed to understand the prognostic implications of these findings.

## Supplementary Information

**Additional file 1.** Additional tables.

## Data Availability

The datasets used and/or analysed during the current study are available from the corresponding author on reasonable request.

## Abbreviations

CAD: Coronary artery disease
CMR: Cardiovascular magnetic resonance
ECV: Extracellular volume fraction
GLS: Global longitudinal strain
LA: Left atrium/left atrial
LGE: Late gadolinium enhancement
LV: Left ventricle/left ventricular
LVEDV: End-diastolic volume
LVEDVI: End-diastolic volume index
LVEF: Left ventricular ejection fraction
LVESV: End-systolic volume
LVESVI: End-systolic volume index
MR: Mitral regurgitation
MS: Mitral stenosis
MVA: Mitral valve area
NT-proBNP: N-terminal pro brain natriuretic peptide
PASP: Pulmonary artery systolic pressure
PHT: Pressure half-time
RA: Right atrium/right atrial
RV: Right ventricle/right ventricular
RVEDV: Right ventricular end-diastolic volume
RVEDVI: Right ventricular end-diastolic volume index
RVEF: Right ventricular ejection fraction
RVESV: Right ventricular end-systolic volume
RVESVI: Right ventricular end-systolic volume index
SV: Stroke volume

## References

[1] Otto CM, Nishimura RA, Bonow RO (2021). 2020 ACC/AHA guideline for the management of patients with valvular heart disease. J Am Coll Cardiol.

[2] Zühlke L, Engel ME, Karthikeyan G (2015). Characteristics, complications, and gaps in evidence-based interventions in rheumatic heart disease: the Global Rheumatic Heart Disease Registry (the REMEDY study). Eur Heart J.

[3] Watkins DA, Johnson CO, Colquhoun SM (2017). Global, regional, and national burden of rheumatic heart disease, 1990–2015. N Engl J Med.

[4] Marijon E, Mocumbi A, Narayanan K, Jouven X, Celermajer DS (2021). Persisting burden and challenges of rheumatic heart disease. Eur Heart J..

[5] Kumar RK, Antunes MJ, Beaton A (2020). Contemporary diagnosis and management of rheumatic heart disease: implications for closing the gap: a scientific statement from the american heart association. Circulation..

[6] Wunderlich NC, Dalvi B, Ho SY, Küx H, Siegel RJ (2019). Rheumatic mitral valve stenosis: diagnosis and treatment options. Curr Cardiol Rep.

[7] Klein AJP, Carroll JD (2006). Left ventricular dysfunction and mitral stenosis. Heart Fail Clin.

[8] Mohan JC, Khalilullah M, Arora R (1989). Left ventricular intrinsic contractility in pure rheumatic mitral stenosis. Am J Cardiol.

[9] Bohbot Y, Renard C, Manrique A (2020). Usefulness of cardiac magnetic resonance imaging in aortic stenosis. Circ Cardiovasc Imaging..

[10] Garg P, Swift AJ, Zhong L (2020). Assessment of mitral valve regurgitation by cardiovascular magnetic resonance imaging. Nat Rev Cardiol.

[11] Zhan Y, Debs D, Khan MA (2020). Natural history of functional tricuspid regurgitation quantified by cardiovascular magnetic resonance. J Am Coll Cardiol.

[12] Djavidani B, Debl K, Lenhart M (2005). Planimetry of mitral valve stenosis by magnetic resonance imaging. J Am Coll Cardiol.

[13] Lin SJ, Brown PA, Watkins MP (2004). Quantification of stenotic mitral valve area with magnetic resonance imaging and comparison with Doppler ultrasound. J Am Coll Cardiol.

[14] Helvacioglu F, Yildirimturk O, Duran C (2014). The evaluation of mitral valve stenosis: comparison of transthoracic echocardiography and cardiac magnetic resonance. Eur Heart J Cardiovasc Imaging.

[15] Cawley PJ, Maki JH, Otto CM (2009). Cardiovascular magnetic resonance imaging for valvular heart disease: technique and validation. Circulation.

[16] Abdelaziz HM, Tawfik AM, Abd-Elsamad AA, Sakr SA, Algamal AM (2020). Cardiac magnetic resonance imaging for assessment of mitral stenosis before and after percutaneous balloon valvuloplasty in comparison to two- and three-dimensional echocardiography. Acta Radiol.

[17] Samaan AA, Said K, El Aroussy W, Hasaan M, Romeih S, El Sawy A, Eid Fawzy M, Yacoub M (2021). Left ventricular remodeling following balloon mitral valvuloplasty in rheumatic mitral stenosis: magnetic resonance imaging study. Front Cardiovasc Med..

[18] Carabello BA (2005). Modern management of mitral stenosis. Circulation.

[19] Levinson GE, Frank MJ, Nadimi M, Braunstein M (1967). Studies of cardiopulmonary blood volume: measurement of left ventricular volume by dye dilution. Circulation.

[20] Wisenbaugh T, Essop R, Middlemost S, Skoularigis J, Sareli P (1992). Excessive vasoconstriction in rheumatic mitral stenosis with modestly reduced ejection fraction. J Am Coll Cardiol.

[21] McKay CR, Kawanishi DT, Kotlewski A (1988). Improvement in exercise capacity and exercise hemodynamics 3 months after double-balloon, catheter balloon valvuloplasty treatment of patients with symptomatic mitral stenosis. Circulation.

[22] Gash AK, Carabello BA, Cepin D, Spann JF (1983). Left ventricular ejection performance and systolic muscle function in patients with mitral stenosis. Circulation.

[23] Kelly DT, Spotnitz HM, Beiser GD, Pierce JE, Epstein SE (1971). Effects of chronic right ventricular volume and pressure loading on left ventricular performance. Circulation.

[24] Shehata ML, Lossnitzer D, Skrok J (2011). Myocardial delayed enhancement in pulmonary hypertension: pulmonary hemodynamics, right ventricular function, and remodeling. Am J Roentgenol.

[25] McCann GP, Gan CT, Beek AM, Niessen HWM, Noordegraaf AV, van Rossum AC (2007). Extent of MRI delayed enhancement of myocardial mass is related to right ventricular dysfunction in pulmonary artery hypertension. Am J Roentgenol.

[26] Sanz J, Dellegrottaglie S, Kariisa M (2007). Prevalence and correlates of septal delayed contrast enhancement in patients with pulmonary hypertension. Am J Cardiol.

[27] Kawel-Boehm N, Hetzel SJ, Ambale-Venkatesh B (2020). Reference ranges (“normal values”) for cardiovascular magnetic resonance (CMR) in adults and children: 2020 update. J Cardiovasc Magn Reson.

