# Supplementary material for: Cardiovascular magnetic resonance characterization of rheumatic mitral stenosis: findings from three worldwide endemic zones
Source: J Cardiovasc Magn Reson. 2022 Apr 7;24:24. doi: 10.1186/s12968-022-00853-5 (PMC8988335; doi:10.1186/s12968-022-00853-5)
Supplement: Supplementary file 1 — Additional file 1. Additional tables. [file 12968_2022_853_MOESM1_ESM.docx]

**Additional file**

Additional file 1: Table S1. Inter-observer and intra-observer variability of CMR measurements.

| **Measurement** | **Inter-observer correlation coefficient** | ***P* value** | **Intra-observer correlation coefficient** | ***P* value** |
| --- | --- | --- | --- | --- |
| MVA | 0.93 | <0.0001 | 0.95 | <0.0001 |
| LA EDVi | 0.98 | <0.0001 | 0.99 | <0.0001 |
| LA ESVi | 0.99 | <0.0001 | 0.99 | <0.0001 |
| RA max area | 0.79 | 0.002 | 0.90 | <0.0001 |
| LV EF | 0.94 | <0.0001 | 0.93 | <0.0001 |
| LV EDVi | 0.89 | <0.0001 | 0.97 | <0.0001 |
| LV ESVi | 0.89 | <0.0001 | 0.97 | <0.0001 |
| RV EF | 0.81 | 0.002 | 0.95 | <0.0001 |
| RV EDVi | 0.81 | 0.002 | 0.98 | <0.0001 |
| RV ESVi | 0.82 | 0.002 | 0.99 | <0.0001 |

CMR, cardiac magnetic resonance; EDVi, end-diastolic volume index; EF, ejection fraction; ESVi, end-systolic volume index; LA, left atrium; LV, left ventricle; MVA, mitral valve area; RA, right atrium; RV, right ventricle.

Additional file 1: Table S2. Correlations between mitral valve area by echocardiography and biatrial and biventricular size/function.

|  | **Correlation coefficient (r)** | ***P* value** |
| --- | --- | --- |
| LA emptying fraction | -0.12 | 0.55 |
| LA maximal volume (mL/m^2^) | 0.14 | 0.48 |
| LA minimal volume (mL/m^2^) | 0.16 | 0.41 |
| LV ejection fraction (%) | 0.04 | 0.82 |
| LV EDV (mL) | 0.30 | 0.12 |
| LV EDVi (mL/m^2^) | 0.21 | 0.27 |
| LV ESV (mL) | 0.20 | 0.31 |
| LV ESVi (mL/m^2^) | 0.13 | 0.52 |
| RV ejection fraction (%) | -0.26 | 0.18 |
| RV EDV (mL) | 0.35 | 0.07 |
| RV EDVi | 0.22 | 0.26 |
| RV ESV (mL) | 0.33 | 0.09 |
| RV ESVi | 0.23 | 0.24 |
| PASP | -0.06 | 0.77 |

EDV, end-diastolic volume; EDVi, End-diastolic volume index; ESV, end-systolic volume; ESVi, end-systolic volume index; LA, left atrium; LV; left ventricle; PASP, pulmonary artery systolic pressure; RA, right atrium; RV, right ventricle.

Additional file 1: Table S3. CMR findings of patients with mitral stenosis (MS), stratified by symptoms

|  | **MS patients without dyspnea on exertion (n=16)** | **MS patients with dyspnea on exertion (n=24)** | ***P* value** |
| --- | --- | --- | --- |
| LA maximal volume (mL/m^2^) | 98 (65, 137) | 84 (68, 103) | 0.22 |
| LA emptying fraction (%) | 18 (11, 34) | 21 (13, 28) | 0.90 |
| RA maximal area (cm^2^) | 21 (17, 25) | 20 (15, 22) | 0.17 |
| LV ejection fraction (%) | 49 (40, 54) | 51 (43, 57) | 0.48 |
| LV EDVi (mL/m^2^) | 78 (67, 93) | 65 (59, 93) | 0.09 |
| RV ejection fraction (%) | 44 (40, 52) | 45 (38, 52) | 0.90 |
| RV EDVi (mL/m^2^) | 72 (65, 80) | 72 (56, 88) | 0.77 |
| LGE presence | (n=15)  13 (87%) | (n=24)  19 (79%) | 0.55 |
| MVA by pressure half-time on echocardiography (cm^2^) | (n=7)  1 (0.8, 1.7) | (n=21)  0.8 (0.7, 1.1) | 0.15 |
|  | **MS patients without palpitations (n=23)** | **MS patients with palpitations (n=17)** | ***P* value** |
| LA maximal volume (mL/m^2^) | 102 (66, 113) | 83 (67, 97) | 0.39 |
| LA emptying fraction (%) | 21 (15, 36) | 17 (11, 27) | 0.21 |
| RA maximal area (cm^2^) | 20 (15, 25) | 20 (16, 22) | 0.54 |
| LV ejection fraction (%) | 51 (41, 55) | 50 (42, 59) | 0.93 |
| LV EDVi (mL/m^2^) | 74 (63, 93) | 66 (57, 92) | 0.27 |
| RV ejection fraction (%) | 44 (40, 52) | 45 (36, 52) | 0.83 |
| RV EDVi (mL/m^2^) | 70 (61, 80) | 74 (56, 89) | 0.58 |
| LGE presence | (n=22)  16 (73%) | (n=17)  16 (94%) | 0.07 |
| MVA by pressure half-time on echocardiography (cm^2^) | (n=13)  0.9 (0.8, 1.2) | (n=15)  0.8 (0.6, 1.1) | 0.76 |
|  | **MS patients without angina (n=22)** | **MS patients with angina (n=18)** | ***P* value** |
| LA maximal volume (mL/m^2^) | 80 (61, 100) | 104 (86, 138) | 0.006 |
| LA emptying fraction (%) | 20 (13, 33) | 19 (12, 28) | 0.77 |
| RA maximal area (cm^2^) | 18 (15, 22) | 22 (19, 25) | 0.04 |
| LV ejection fraction (%) | 50 (42, 54) | 51 (41, 63) | 0.39 |
| LV EDVi (mL/m^2^) | 64 (59, 87) | 78 (66, 93) | 0.09 |
| RV ejection fraction (%) | 43 (37, 50) | 44 (40, 55) | 0.37 |
| RV EDVi (mL/m^2^) | 76 (68, 89) | 68 (55, 80) | 0.07 |
| LGE presence | (n=21)  17 (81%) | (n=17)  14 (82%) | 0.91 |
| MVA by pressure half-time on echocardiography (cm^2^) | (n=20)  0.8 (0.73, 1) | (n=8)  1.15 (0.65, 1.65) | 0.18 |

Data are shown as median (interquartile range) or n (%). CMR, cardiac magnetic resonance; EDVi, End-diastolic volume index; LA, left atrium; LGE, late gadolinium enhancement; LV, left ventricle; MS, mitral stenosis; MVA, mitral valve area; RA, right atrium; RV, right ventricle.

Additional file 1: Table S4. CMR findings of the mitral stenosis (MS) cohort, stratified by presence of LGE

|  | **MS patients without LGE (n=7)** | **MS patients with LGE (n=32)** | ***P* value** |
| --- | --- | --- | --- |
| LA maximal volume (mL/m^2^) | 97 (59, 124) | 86 (68, 108) | 0.92 |
| LA emptying fraction (%) | 19 (14, 33) | 20 (13, 30) | 0.89 |
| RA maximal area (cm^2^) | 19 (10, 25) | 20 (17, 23) | 0.63 |
| LV ejection fraction (%) | 48 (41, 53) | 51 (43, 57) | 0.37 |
| LV EDV (mL) | 114 (81, 152) | 112 (100, 154) | 0.65 |
| LV EDVi (mL/m^2^) | 74 (58, 83) | 69 (61, 93) | 0.76 |
| LV ESV (mL) | 48 (43, 79) | 57 (43, 78) | 0.66 |
| LV ESVi (mL/m^2^) | 33 (27, 43) | 35 (27, 49) | 0.78 |
| RV ejection fraction (%) | 51 (40, 52) | 44 (38, 52) | 0.57 |
| RV EDV (mL) | 100 (73, 130) | 113 (99, 142) | 0.39 |
| RV EDVi (mL/m^2^) | 68 (53, 76) | 73 (59, 88) | 0.33 |
| RV ESV (mL) | 60 (36, 73) | 67 (47, 80) | 0.31 |
| RV ESVi (mL/m^2^) | 35 (26, 43) | 41 (29, 50) | 0.35 |
| MVA by pressure half-time on echocardiography (cm^2^) | (n=3)  0.8 (0.6, 0.8) | (n=25)  0.9 (0.8, 1.2) | 0.18 |

Data are shown as median (interquartile range). CMR, cardiac magnetic resonance; EDV, end-diastolic volume; EDVi, End-diastolic volume index; ESV, end-systolic volume; ESVi, end-systolic volume index; LA, left atrium; LGE, late gadolinium enhancement; LV, left ventricle; MS, mitral stenosis; RA, right atrium; RV, right ventricle.

Additional file 1: Table S5. Clinical characteristics and CMR findings of patients who underwent a mitral valve procedure for mitral stenosis.

| **Characteristics** | **Valve procedure (n=22)** | **No valve procedure (n=16)** | ***P* value** |
| --- | --- | --- | --- |
| Age | 52 (37, 58) | 39 (23, 47) | 0.02 |
| Female | 18 (82%) | 10 (63%) | 0.18 |
| Atrial fibrillation | 11 (50%) | 5 (31%) | 0.24 |
| Diabetes | 2 (9%) | 1 (6%) | 0.75 |
| Hypertension | 3 (14%) | 2 (13%) | 0.92 |
| Dyspnea on exertion | 14 (64%) | 10 (63%) | 0.94 |
| Syncope | 1 (5%) | 2 (13%) | 0.37 |
| Palpitations | 11 (50%) | 6 (38%) | 0.44 |
| Angina | 14 (64%) | 3 (19%) | 0.006 |
| LA emptying fraction (%) | 21 (13, 29) | 17 (13, 32) | 0.54 |
| LA EDVi (mL/m^2^) | 97 (73, 127) | 80 (59, 103) | 0.16 |
| RA maximal area (cm^2^) | 20 (17, 23) | 20 (15, 22) | 0.90 |
| MVA by pressure half-time on echocardiography (cm^2^) | 0.8 (0.75, 1.1) | 0.95 (0.75, 1.2) | 0.50 |
| MVA by planimetry on CMR (cm^2^) | 1.32 (0.97, 1.68) | 1.08 (0.86, 1.25) | 0.18 |
| LV ejection fraction (%) | 52 (43, 61) | 47 (40, 53) | 0.13 |
| LV EDVi (mL/m^2^) | 74 (60, 93) | 74 (60, 93) | 0.94 |
| LV global longitudinal strain (%) | (n=11)  -17 (-18, -16) | (n=9)  -15 (-16, -13) | 0.03 |
| RV ejection fraction (%) | 44 (39, 52) | 44 (36, 52) | 0.73 |
| RV EDVi (mL/m^2^) | 69 (55, 78) | 84 (68, 94) | 0.02 |
| LGE presence | (n=22)  17 (77%) | (n=15)  13 (87%) | 0.47 |
| Extracellular volume (%) | (n=9)  26.8 (22.2, 29.5) | (n=8)  28.3 (23.3, 30.9) | 0.70 |

Data are shown as median (interquartile range) or n (%). CMR, cardiac magnetic resonance; EDVi, End-diastolic volume index; LA, left atrium; LGE, late gadolinium enhancement; LV, left ventricle; MVA, mitral valve area; RA, right atrium; RV, right ventricle.

Additional file 1: Table S6. Clinical characteristics and CMR findings of patients who underwent a mitral valve procedure for mitral stenosis, stratified by type of valve procedure.

| **Characteristics** | **Valve Replacement (n=14)** | **Valvuloplasty**  **(n=8)** | ***P* value** |
| --- | --- | --- | --- |
| LA emptying fraction (%) | 21 (11, 29) | 22 (14, 38) | 0.64 |
| LA EDVi (mL/m^2^) | 102 (84, 153) | 78 (64, 102) | 0.06 |
| RA maximal area (cm^2^) | 22 (18, 24) | 17 (14, 21) | 0.08 |
| LV ejection fraction | 55 (41, 64) | 50 (44, 54) | 0.32 |
| LV EDVi (mL/m^2^) | 85 (66, 98) | 64 (59, 67) | 0.07 |
| LV global longitudinal strain (%) | (n=4)  -17 (-19, -16) | (n=7)  -16 (-18, -14) | 0.64 |
| RV ejection fraction (%) | 45 (40, 55) | 41 (37, 48) | 0.18 |
| RV EDVi (mL/m^2^) | 63 (53, 76) | 73 (68, 85) | 0.18 |
| LGE presence | (n=14)  11 (79%) | (n=8)  6 (75%) | 0.85 |

Data are shown as median (interquartile range) or n (%). CMR, cardiac magnetic resonance; EDVi, End-diastolic volume index; LA, left atrium; LGE, late gadolinium enhancement; LV, left ventricle; MVA, mitral valve area; RA, right atrium; RV, right ventricle.
